# Sets of serum exosomal microRNAs as candidate diagnostic biomarkers for Kawasaki disease

**DOI:** 10.1038/srep44706

**Published:** 2017-03-20

**Authors:** Hong-Ling Jia, Chao-Wu Liu, Li Zhang, Wei-Jun Xu, Xue-Juan Gao, Jun Bai, Yu-Fen Xu, Ming-Guo Xu, Gong Zhang

**Affiliations:** 1 Key Laboratory of Functional Protein Research of Guangdong Higher Education Institutes, Institute of Life and Health Engineering, College of Life Science and Technology, Jinan University, Guangzhou 510632, Guangdong, China; 2 State Key Laboratory of Applied Microbiology Southern China, Guangdong Provincial Key Laboratory of Microbial Culture Collection and Application, Guangdong Institute of Microbiology, Guangzhou 510070, Guangdong, China; 3 Guangzhou Women and Children’s Medical Center, Guangzhou 510120, Guangdong, China; 4 Information Center, Shenzhen Children’s Hospital, Shenzhen, 518038, Guangdong, China; 5 Foshan Women and Children’s Hospital, Foshan, 528000, Guangdong, China; 6 Department of Pediatric Cardiology, Shenzhen Children’s Hospital, Shenzhen, 518038, Guangdong, China

## Abstract

Although Kawasaki disease is the main cause of acquired heart disease in children, no diagnostic biomarkers are available. We aimed to identify candidate biomarkers for diagnosing Kawasaki disease using serum exosomal microRNAs (miRNAs). Using frozen serum samples from a biobank, high-throughput microarray technologies, two-stage real-time quantitative PCR, and a self-referencing strategy for data normalization, we narrowed down the list of biomarker candidates to a set of 4 miRNAs. We further validated the diagnostic capabilities of the identified miRNAs (namely, C_T_(miR-1246)-C_T_(miR-4436b-5p) and C_T_(miR-197-3p)-C_T_(miR-671-5p)) in 79 samples from two hospitals. We found that this 4-miRNA set could distinguish KD patients from other febrile patients as well as from healthy individuals in a single pass, with a minimal rate of false positives and negatives. We thus propose, for the first time, that serum exosomal miRNAs represent candidate diagnostic biomarkers for Kawasaki disease. Additionally, we describe an effective strategy of screening for biomarkers of complex diseases even when little mechanistic knowledge is available.

Kawasaki disease (KD) is characterized by acute, febrile rash mainly occurring among children younger than 5 years and resulting in coronary arterial lesions. KD is becoming the leading cause of acquired heart diseases in children^1^,^2^. To date, there is no specific diagnostic test for KD, and the diagnosis is still highly dependent on the symptoms manifested and on ultrasonic imaging findings^3^. Intravenous immunoglobulin (IVIG) injection and aspirin therapy have been applied as an effective treatment against KD, reducing the rate of complications involving the coronary artery from about 25% to 5%^4^. Some cases of KD are easily misdiagnosed as respiratory tract infections, septicaemia, epistaxis, scarlet fever (SF), bovillae, lymphadenitis, or juvenile idiopathic arthritis (JIA)^5^. Delayed treatment often leads to a high risk of coronary artery damage.

Proteomics technology has been applied to screen serum protein biomarkers of KD^6^. However, the most significant differentially expressed proteins are components of the immune response, such as transthyretin (TTR), complement C3 and serum albumin (ALB), or proteins indicative of arterial lesions, such as fibrinogen^7^ and cardiac troponin I^8^. Our previous investigation revealed that the levels of serum proteins such as TTR can reflect the effectiveness of IVIG treatment, which can serve as a sensitive marker for IVIG treatment response^6^. However, they are not effective for diagnosis of KD^8^.

Indeed, proteomic methods have their intrinsic limitations, which have been encountered in the Chromosome-centric Human Proteome Project. These limitations include low sensitivity to quantify low-abundant proteins at a practical cost, dependence on the physical and chemical properties of proteins, low peptide sequence coverage, difficulty in detecting single amino acid variations, inefficiency of new protein discovery and “low-staining problem” of antibody-based detection^9^,^10^. Because of these drawbacks, protein biomarkers tend to have a higher rate of false positives and false negatives than those noted for nucleic acid biomarkers in clinical diagnosis (for example, in the prenatal diagnosis of trisomy^11^,^12^,^13^).

Among the various types of nucleic acids, microRNA (miRNA) has been regarded as a promising resource of biomarkers. miRNAs represent a category of functional single-stranded small RNAs ranging from 17 to 25 nucleotides in length. miRNAs mediate the translational control of target genes and play an important role in the regulation of physiological and pathological processes^14^,^15^,^16^,^17^. It is estimated that 60–70% of the human coding genes are regulated by miRNAs^18^. In 2008, Mitchell *et al*. detected 37 miRNAs from the plasma samples of healthy individuals, including let-7a, miR-16 and miR-15b^19^. They found that miRNAs can exist in a stable form in the plasma, escaping RNase degradation. Using high-throughput sequencing techniques, more than 100 miRNAs have been identified from human serum^20^. miRNA profiles are specific to various physiological and pathological conditions, wherein lies their potential as diagnostic biomarkers^21^.

Recent studies discovered that miRNAs residing in microvesicles are protected from RNase degradation. Large microvesicles originate from apoptotic and necrotic cells, creating a high background of encapsulated miRNA,and their levels vary significantly with physiological and pathological conditions^22^. Shimizu *et al*. screened six differentially expressed miRNAs isolated from microvesicles obtained from KD patients, but found that none of these miRNAs could distinguish KD from adenovirus infection, suggesting that miRNAs from large microvesicles are not sufficiently specific for KD diagnosis^23^.

Exosomes, which are another type of small microvesicles with a diameter of 30–100 nm, are selectively and actively packed and secreted by most live cells, containing multiple types of nucleic acids and various protein species^24^,^25^. The functional nucleic acids and proteins packed in the exosomes can be imported into target cells, regulating gene expression. This suggests that exosomes serve as active media of intercellular communication and thus play important roles in physiological and pathological processes^22^,^26^,^27^. Therefore, exosomes are considered to be a potential source of diagnostic biomarkers. Indeed, it has been shown that exosome miRNAs can be useful in the early diagnosis of cancer^20^.

High-throughput methods can be efficient for screening potential diagnostic miRNA biomarkers. However, numerous additional challenges have to be thoroughly considered when designing such studies, including pre-analytic variation and data normalization^28^. Normalization and standard selection of circulating miRNAs represents a complicated task. For this reason, most available models were deduced based on background gene expression profiles in the cells^29^,^30^,^31^, which is completely absent in exosomes.

In order to screen miRNAs potentially helpful for KD diagnosis, we compared the profiles of exosomal miRNAs extracted from the serum of KD patients and that of case controls. We used a computational approach to select pairs of miRNAs that can serve as diagnostic biomarkers, to solve the problem of internal standard selection and to improve robustness. These pairs of miRNAs could distinguish KD from infectious diseases and JIA, which share similar symptoms with KD, suggesting that such biomarkers may be clinically applicable for the diagnosis of KD.

## Results

### Exosome isolation and validation

**Figure 1:**
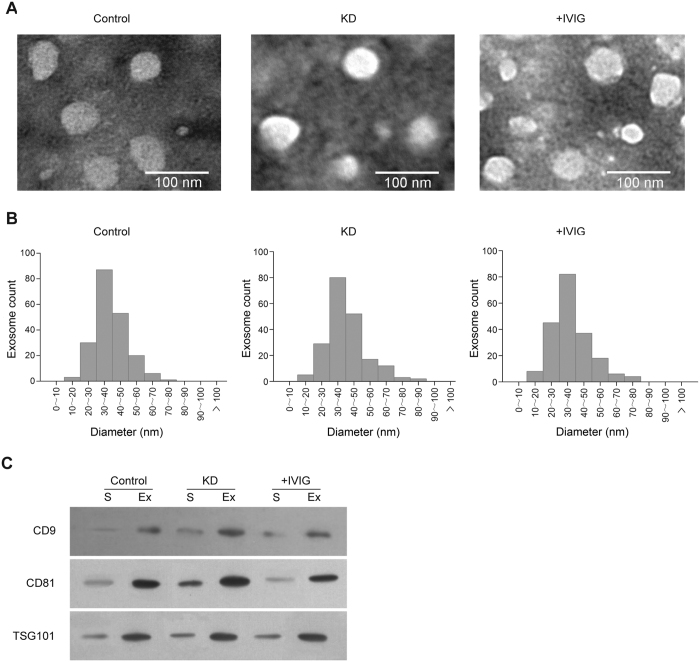
Characterization of serum exosomes. (**A**) Typical transmission electron microscopy images of serum exosomes isolated from healthy controls (Normal), from Kawasaki disease patients (KD), and from Kawasaki disease patients after intravenous immunoglobulin treatment (+IVIG). (**B**) Statistics regarding vesicle diameters for the three sample types in (**A**), measured based on the transmission electron microscopy images. For each type of sample, 200 vesicles were measured. (**C**) Western blot of exosome protein markers (CD9, CD81, and TSG101) in total serum and isolated exosomes. Equal amounts of protein were loaded in each lane.

We first assessed the exosomes isolated from the serum. Transmission electron microscopy (TEM) revealed spherical vesicles approximately 30–100 nm in diameter in all samples (Fig. 1A), which is consistent with previously reported characteristics of exosomes. No microvesicle with the diameter larger than 100 nm was detected (Fig. 1B), suggesting that we had sufficiently purified exosomes from the serum. We performed western blotting to detect three commonly used exosomal protein markers, namely CD9, CD81 and TSG101^32^,^33^. With an equal amount of proteins loaded on each lane, the three exosome protein markers were all highly enriched in the isolated exosomes relative to the serum (Fig. 1C). These results confirmed that we have successfully purified entire exosomes from all serum samples.

### Differential exosomal miRNA profiling to screen miRNA biomarker candidates

**Figure 2:**
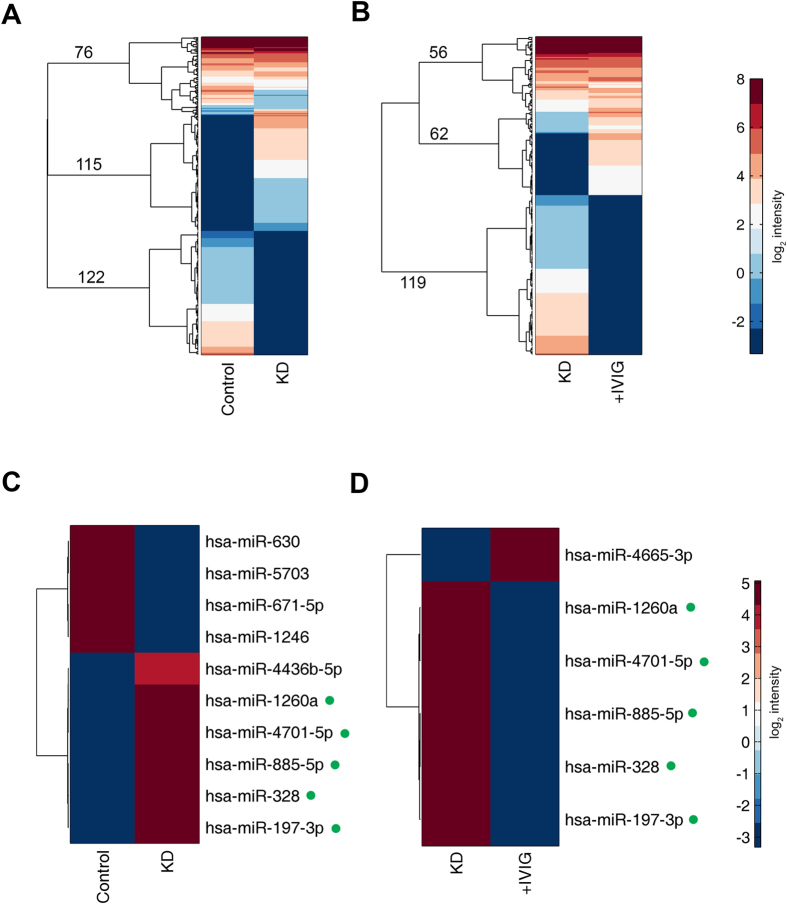
Microarray detection of exosome miRNA. (**A**,**B**) Clustergram of miRNA signal intensities of data from healthy controls versus data from Kawasaki disease patients (**A**); and of data from Kawasaki disease patients versus data from Kawasaki disease patients who underwent immunoglobulin treatment (**B**). miRNA for whose Log_2_ intensity <−3 (darkest blue in the colour scale) were considered undetected. Clustering was performed using Euclidean distances and Ward’s linkage. The numbers represent the number of miRNAs in each main branch among the top three branches of the dendrogram. (**C**,**D**) Selected miRNAs from (**A**,**B**) who exhibited a difference in expression >200-fold. The five miRNAs with green dots after their names were exhibited a difference in expression >200-fold.

A total of 300 ng of RNA could be yielded from 400 μl of serum. We used miRNA microarray to screen for significantly changed miRNAs in KD patients, before and after IVIG treatment. The fluorescent signals of the miRNAs detected in the microarray were compared between samples from healthy volunteers and those from patients with KD (Fig. 2A), as well as between samples from patients with KD obtained before and after IVIG treatment (Fig. 2B). For both comparisons, only a small fraction of miRNAs (76 and 56, respectively) were detected with relatively high expression (log_2_ intensity >1)in both samples being compared. Most of the miRNAs (237 and 181, respectively) exhibited a large difference between the two samples being compared, and most showed an “on/off” switch. These results reflect a dramatic change in small RNAs in the somatic cells, indicating that such miRNAs can likely serve as a pool for biomarker candidates.

We then selected the miRNAs with dramatic changes (>200-fold) in both microarrays (i.e., healthy volunteers vs. KD patients, and KD patients before and after IVIG treatment; Fig. 2C and D, respectively). All these miRNAs showed a strict on/off switch: high signal in one sample (log_2_ intensity >3.5) and undetected in another (log_2_ intensity <−3, which is below the detection limit of the microarray). Of note, five miRNAs, namely miR-1260a, miR-4701-5p, miR-885-5p, miR-328 and miR-197-3p, were also detected as having significant change in both microarrays, but with opposite tendency; specifically, they were detected with high abundance (log_2_ intensity >4.5) in KD patients, and remained undetectable in the healthy individuals and IVIG-treated patients. These findings indicated that the identified miRNAs may be closely correlated with KD.

### Internal reference selection by pair-wise statistical tests on quantitative reverse-transcription PCR (qRT-PCR) results

**Figure 3:**
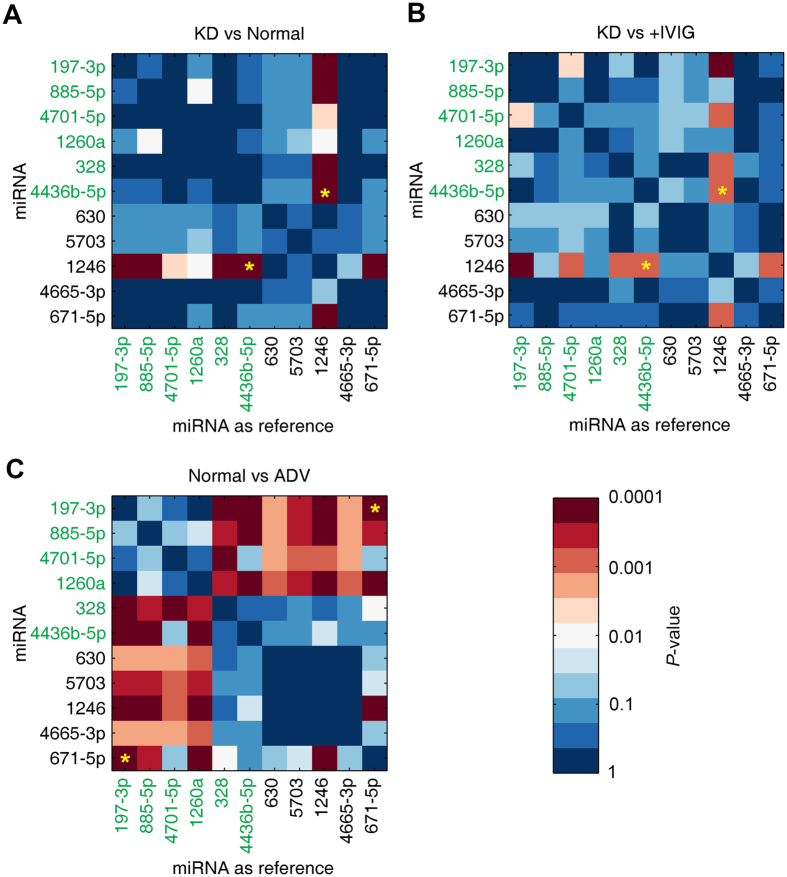
Pair-wise t-test for all candidate miRNAs using each miRNA as internal reference. A cohort of 20 healthy individuals (Normal), 20 Kawasaki disease patients (KD), 20 Kawasaki disease patients after IVIG treatment (+IVIG), and 5 adenovirus-infected patients without Kawasaki disease (ADV) was examined for 11 candidate miRNAs using qRT-PCR. The *P*-values of the t-tests between each pair of groups were shown in a color scale. miRNAs in green fonts represent candidate miRNAs that are highly expressed only in Kawasaki disease patients but are not detected in healthy individuals or in Kawasaki disease patients who underwent immunoglobulin injection treatment (Fig. 2C and D). (**A**) KD group vs. Normal group. The yellow stars denote the final selected pairs. (**B**) KD group vs. +IVIG group; (**C**) Normal group vs. ADV group.

To validate the microarray results and further narrow down the list of biomarkers, we performed qRT-PCR to quantify the 11 candidate miRNAs identified above for an independent cohort of 60 samples obtained from a biobank of frozen samples. This cohort included samples from 20 healthy individuals, 20 KD patients before IVIG treatment and 20 KD patients after IVIG treatment. To test the specificity of the potential biomarkers, we also included 5 patients infected with adenovirus (ADV). Our decision was motivated by the fact that KD is easily misdiagnosed as an infectious disease caused by ADV or similar agents, associated with similar febrile symptoms. We expect that the diagnostic efficiency of trace amounts of exosomal miRNAs may vary significantly, especially in the clinical setting. Therefore, an internal reference is essential for accurate quantification. However, the internal reference is not available in the case of exosomes because of the lack of *a priori* knowledge. To overcome this obstacle, we performed pair-wise analysis of qRT-PCR results, trying each possible miRNA as an internal reference. Between-group differences in miRNA expression levels were statistically examined using the t-test. The P-values were visualized using matrices with a colour scale (Fig. 3).

When using this larger cohort of 60 samples, most miRNA pairs showed less significance compared to that of microarray findings regarding the difference between KD and healthy controls, with most P-values > 0.01 (Fig. 3A). As IVIG is an effective treatment against KD, we expected a similar pattern of differential expression (versus controls) in IVIG-treated patients with KD, which was confirmed by our data (Fig. 3B).

Potential biomarkers should ideally meet the following criteria: (a) significantly different between KD and healthy controls (P < 0.01, Fig. 3A); (b) significantly different between KD and IVIG-treated KD (P < 0.01, Fig. 3B); (c) not significantly different between healthy controls and ADV-infected patients (P > 0.01, Fig. 3C). The pair miR-1246/miR-4436b-5p was the only biomarker that fulfilled all criteria. An additional pair, miR-197-3p/miR-671-5p, showed a completely opposite trend for the above-mentioned criteria, suggesting that it may serve as an additional biomarker to distinguish viral infection from KD. Indeed, miR-197 is a predictor for death in symptomatic coronary artery disease^34^, whereas circulating miR-1246 has been shown to indicate diastolic dysfunction^35^. These findings imply that our selected miRNAs may reflect cardiovascular lesions in KD.

### Validation of the potential biomarkers for KD diagnosis

**Figure 4:**
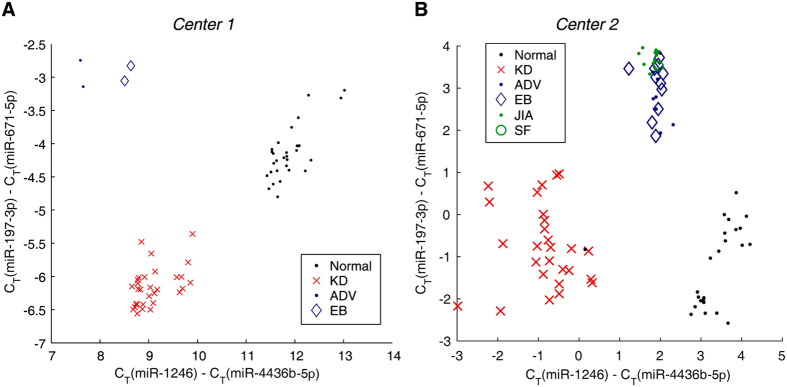
Kawasaki disease diagnostic test on two centres (panel A and B). Normal individuals (Normal, black dots), Kawasaki disease patients (KD, red cross), adenovirus-infected patients (ADV, blue dots), patients infected with epstein-barr virus (EB, blue diamonds), patients with juvenile idiopathic arthritis (JIA, green dots), and patients with scarlet fever (SF, green circle). The C_T_ values of the two pairs of candidate miRNA biomarkers are shown. The diagnostic test carried out in Centre 2 was single-blinded.

To test the diagnostic capability of the potential biomarkers identified in our study, we collected fresh blood samples from two hospitals. The validation cohort included 54 healthy individuals, 54 KD patients who had not received IVIG therapy, 10 ADV patients, 13 epstein-barr virus (EBV) patients, 12 JIA patients and 1 SF patient. Validation was performed as a normal test in one hospital (Centre 1) and as a single-blind test in the other hospital (Centre 2). We plotted the qRT-PCR quantification results of the two pairs of miRNAs for all samples in the validation cohort (see scatter plot in Fig. 4). When analysing both pairs of miRNAs, healthy individuals, KD patients and non-KD patients can be clearly separated as three clusters, with only one exception (visible for samples from Centre 2). These findings indicated good diagnostic capabilities, at least in the cohorts examined, suggesting that these two-pairs of miRNAs can be used as biomarkers for KD. Of note, neither pair of miRNAs can independently distinguish KD patients from the healthy individuals and those with viral infection, highlighting the need to combine multiple miRNA pairs.

## Discussion

In this study, we established a method to screen KD-specific miRNAs from serum exosomes. Although exosomal miRNAs were proposed as a potential resource of biomarkers several years ago, little has been investigated and implemented in clinical practice for the management of cardiovascular diseases^36^. To our knowledge, this is the first study that successfully obtained two pairs of exosomal miRNAs from a mass of serum miRNAs to be used as biomarkers for KD.

Technically, when comparing to previous miRNA-based studies of KD, which only yielded 11% of the exosomes^23^, we have isolated serum exosomes with high and stable purity, ensuring a reliable miRNA source. We also obtained microarray signals for such trace amounts of exosomal miRNA, which allowed us to assess all known miRNAs without *a priori* knowledge of relevant pathways. This unbiased approach is especially beneficial for managing diseases with unknown aetiology, such as KD.

Strategically, we first selected the groups of miRNAs with on/off behaviour for KD versus healthy controls and for KD versus IVIG-treated patients^6^. This considerably narrowed down the number of candidate miRNAs, from several thousands to 11, which allowed individual qRT-PCR validation and further screening on a practical scale. When we performed the final validation, we included febrile controls with ADV and EBV infections. Furthermore, we also recruited 12 patients with JIA and 1 patient with SF to be included in the control group; in these patients, clinical findings and laboratory results are similar to those of patients with KD. Although the sample size is limited, which may reduce the statistical power as a limitation of this study, this approach is more effective than previous strategies of validating candidate biomarkers in febrile controls with viral infection. In the present study, five pairs of miRNAs met the criteria of *P* < 10^−4^for the comparison between patients with KD and healthy volunteers, but only one remained significant when we also considered the comparison between KD and viral infection. Meanwhile, the latter comparison alone (i.e., KD versus viral infections) also provided a reverse miRNA pair, which enhanced the specificity.

Notably, we applied a strategy of normalization that involved considering each miRNA as reference for all other miRNAs (Fig. 3), which is computationally intensive. However, this solved a major problem especially in the analysis of samples with trace amounts of target miRNA, since the extraction efficiency is highly variable. Such self-referencing cancels errors arising during sample processing, which is a much more robust approach than comparing the results of patients against those of healthy volunteers, and is very useful in the real clinical setting because it reduces the requirement for high accuracy of manipulations. Additionally, using multiple criteria instead of a single one should be effective in KD diagnosis, since the aetiology of KD remains unclear, and several molecular pathways may be related to the onset and development of KD. Our results confirm this expectation, as it was necessary to use two miRNA pairs as a combined criterion for achieving good diagnostic capabilities. This strategy differs from that applied in most previous studies, which used a single criterion for simple diagnosis. Applying the self-referencing scheme and using multiple criteria allowed us to yield a stable performance of biomarker quantification, minimizing the rate of false positives and false negatives, at least as confirmed in our validation cohort.

We have validated these miRNA-based biomarkers in a multi-centre test. However, it should be noted that the cycle threshold (C_T_) values were different for the samples collected from the two hospitals, which is related to the use of different instruments and reagents. In future clinical applications, a set of standard samples should be included to calibrate centre-specific reference ranges.

Little is known about the functions of these miRNAs, especially in the context of cardiovascular diseases. The present study provides insights regarding the relevance of these miRNAs in KD, especially regarding the response to lesions of vascular endothelial cells, which is worthy of further mechanistic investigations. Nevertheless, because the present study focused on the potential application of miRNAs as diagnostic biomarkers, mechanistic information is not essential.

This study also revealed valuable information regarding the viability of frozen serum samples from biobank, which can serve as a precious resource of biomarker discovery, at least with respect to exosomal miRNA. In this study, we started our investigation using frozen samples, and were able to validate the exosomal miRNA biomarker candidates using fresh samples, which indicates that the presented strategy is highly effective. Our self-referencing scheme is a major contributor to this robustness, because self-referencing cancels the response fluctuations associated with sample storage and processing.

While certain limitations should be considered, our present study not only provided a set of serum exosomal miRNA-based biomarkers for diagnosis KD, but also demonstrated a strategy of screening effective biomarkers for complex diseases even when little mechanistic knowledge is available, taking advantage of the rich source of biobank materials.

## Methods

### Preparation of serum samples

Blood samples from 79 KD patients who did not undergo IVIG therapy and 25 KD patients who did undergo IVIG therapy were randomly selected.All KD patients met the diagnostic criteria for KD established by the American Heart Association and the Japanese Ministry of Health and Welfare criteria^37^,^38^,^39^.The clinical diagnostic data are listed in the Supplementary File (Tables S1–S4).

First, we obtained the frozen samples from the biobank of Guangzhou Women and Children’s Medical Center (Centre 1) and Shenzhen Children’s Hospital (Centre 2), including a random selection of samples from 10 KD patients before and after IVIG therapy, from 15 KD patients before IVIG therapy and from another 15 KD patients after IVIG therapy, from 25 healthy children, and from 5 patients with ADV infection to be used for microarray analysis and validation of screening results.

Second, we obtained fresh samples from a random selection of 30 KD patients before IVIG therapy, 2 ADV patients, 2 EBV patients and 30 healthy children from Guangzhou Women and Children’s Medical Center, as well as fresh samples from 24 KD patients before IVIG therapy,8 ADV patients,11 EBV patients,12 JIA patients,1 SF patient and 24 healthy children randomly enrolled from Shenzhen Children’s Hospital for validation of candidate miRNAs biomarkers.Approval for the collection of human samples was approved by the Ethics Committees at Guangzhou Women and Children’s Medical Center (approval number [2013]077), and written informed consent was obtained from all guardians.

Serum samples were separated by centrifugation at 1,000 × g for 10 min. Serum aliquots were collected and stored at −80 °C. The serum obtained was further processed for exosome isolation.

### ExoQuick extraction of serum exosomes

We isolated exosomes from the serum of all participants by using ExoQuick precipitation (System Biosciences Inc, Mountain View, CA) following the manufacturer’s instructions^40^,^41^. In brief, the exosomes were pelleted by adding the exosome extraction reagent and centrifuging at 1,500 × g for 10 min at 4 °C. The exosome pellet was resuspended in 10 mM PBS in four times the volume of serum.

### Exosome characterization

#### TEM

A copper mesh was placed on a clean wax plate and 100 μl of the exosome suspension was added. After 4 minutes, the copper mesh was removed and placed in 2% phosphotungstic acid for 5 min. The mesh was laid on the filter paper to dry and TEM was used to observe the morphological features of the exosome.

#### Western blot analysis

The exosome pellet was dissolved in the protein lysis buffer, and the protein concentration was determined using a Bradford protein assay kit (Bio-Rad Laboratories, Hercules, CA). The proteins were separated on an SDS-PAGE gel before transferring to a PVDF membrane. The membrane was incubated with TSG101, CD9 and CD81 primary antibodies at 4 °C overnight, followed by incubation with the corresponding secondary antibodies at room temperature for 1 h. Specific protein bands were visualized using the SuperSignal chemiluminescence system (ECL, Pierce, USA) and imaged by X-ray film.

### RNA extraction from exosomes

RNA was extracted from the exosome pellets using a Trizol reagent (Invitrogen Life Technologies, Carlsbad, CA) according to the manufacturer’s protocol. Briefly, 1.0 ml of Trizol reagent and 200 μl of chloroform were added to the sample, and the mixture was vortexed for 60 s and allowed to stand at 25 °C for 5 min. After the mixture was centrifuged at 10,000 × g for 10 min at 4 °C, the supernatant was transferred to a fresh tube and 500 μl of isopropanol were added. After overnight incubation at −20 °C, the mixture was centrifuged at 10,000 × g for 10 min at 4 °C to remove the supernatant, and the RNA pellet was washed with 75% ethanol. The RNA pellet was dissolved in 20 μl of RNase-free water. The purity of the isolated RNA was determined according to the ratio of radiation absorbance at 260 nm to that at 280 nm, using a Nanodrop ND-1000 system (Thermo Fisher Scientific, South San Francisco, CA).

### Microarray analysis

Exosomes extracted from equal amounts of serum from three sources (5 healthy patients, 5 KD patients before IVIG therapy, and 5 KD patients after IVIG therapy) were pooled into three individual samples and used for miRNA microarray analysis. Total miRNA from these three pooled samples was extracted as described in the previous paragraph. Microarray hybridization, data generation, and normalization were performed by Shanghai Biochip Corp. according to standard Agilent protocols. Human miRNA microarrays from Agilent Technologies, which contain probes of 1,887 human miRNAs from the Sanger database v.18.0, were used in this study. Visualization of microarray data was performed using MeV 4.6 software (MultiExperiment Viewer; http://www.tm4.org/mev/). A miRNA was considered overexpressed if expression in one of the pooled samples was >200-fold higher than that in another pooled sample. The overexpressed miRNAs were considered candidate miRNA biomarkers for further analysis. The microarray data are available in Gene Expression Omnibus with the accession number GSE60965.

### Validation of the qRT-PCR results

To validate the microarray data, a qRT-PCR experiment was performed using the Power SYBR Green PCR Master Mix (Applied Biosystems, Foster City, CA) in an ABI 7500 Real-Time PCR System (Applied Biosystems). The assays were performed on 60 samples (20 healthy patients, 20 KD patients before IVIG therapy, and 20 KD patients after IVIG therapy) for 4 miRNAs (miR-4436b-5p, miR-1246, miR-671-5p, and miR-197-3p) that met the defined criteria. Each reaction was performed in a 20-μl volume system containing 5 μl of cDNA, 0.5 μl of each primer, 10 μl of Power SYBR Green PCR Master Mix, and 4.0 μl of RNase-free water. The PCR program consisted of denaturation at 95 °C for 2 min, followed by 40 cycles consisting of denaturation for 15 s at 95 °C and annealing and extension for 30 s at 60 °C. All operations were conducted in triplicate. miRNAs that exhibited cycle threshold values above 35 in any of the 60 samples were excluded from additional statistical analyses.

To test the diagnostic capabilities of the identified miRNAs for KD, a qRT-PCR-based evaluation of miR-4436b-5p, miR-1246, miR-671-5p, and miR-197-3p expression was performed following the same steps described in the previous paragraph for patients with ADV, EBV, JIA, or SF infection.

All RT-PCR primers used in this study are listed in Supplementary Table S5.

### Statistical analysis

Since an internal standard for normalization was not available, we performed normalization using each candidate miRNA as reference. Statistical significance was determined using Student’s t-test, and a *P* < 0.01 was considered to indicate statistical significance.

We confirm that all experiments were performed in accordance with approved guidelines and regulations by Jinan University and Guangzhou Women and Children’s Medical Center. All experimental protocols were approved by the Ethics Committees at Guangzhou Women and Children’s Medical Center. Written informed consent was obtained from all participants in accordance with the Declaration of Helsinki.

## Additional Information

**How to cite this article:** Jia, H.-L. *et al*. Sets of serum exosomal microRNAs as candidate diagnostic biomarkers for Kawasaki disease. *Sci. Rep.* **7**, 44706; doi: 10.1038/srep44706 (2017).

**Publisher's note:** Springer Nature remains neutral with regard to jurisdictional claims in published maps and institutional affiliations.

## Supplementary Material

Supplementary Material
