# Supplementary material for: Sets of serum exosomal microRNAs as candidate diagnostic biomarkers for Kawasaki disease
Source: Sci Rep. 2017 Mar 20;7:44706. doi: 10.1038/srep44706 (PMC5357789; doi:10.1038/srep44706)
Supplement: Supplementary Material [file srep44706-s1.pdf]

Sets of serum exosomal microRNAs as candidate diagnostic biomarkers for Kawasaki disease

Hong-Ling Jia[1,†], Chao-Wu Liu[3,†], Li Zhang[2,*],Wei-Jun Xu[5,†], Xue-Juan Gao[1], Jun Bai[4], Yu-Fen Xu[2], Ming-Guo Xu[6,*] and Gong Zhang[1,*]

Table S1

Coronary artery changes in 5 untreated and 5 intravenous immunoglobulin-treated Kawasaki disease patients (these samples were used for miRNA microarray analysis）

Patient	Age	Sex	Treatment	Coronary Change
Patient 5	6M	male	- IVIG	LCA=3.5 mm RCA=2.7 mm
Patient 14	1Y10M	female	- IVIG	LCA=3.3 mm RCA=3.0 mm
Patient 19	1Y5M	female	- IVIG	LCA=3.2 mm RCA=2.3 mm
Patient 34	1Y7M	male	- IVIG	LCA=3.1 mm RCA=3.2 mm
Patient 43	7M	male	- IVIG	LCA=2.4 mm RCA=3.0 mm
Patient 4	1Y4M	male	+ IVIG	LCA=2.8 mm RCA=2.0 mm
Patient 10	2Y1M	female	+ IVIG	LCA=2.5 mm RCA=2.8 mm
Patient 30	1Y5M	female	+ IVIG	LCA=3.0 mm RCA=2.3 mm
Patient 35	1Y9M	male	+ IVIG	LCA=2.3 mm RCA=3.0 mm
Patient 40	5M	male	+ IVIG	LCA=2.2 mm RCA=2.4 mm

Y = year; M = month; IVIG = intravenous immunoglobulin; -IVIG = KD patients before IVIG therapy; +IVIG = KD patients after IVIG therapy; LCA = left coronary artery; RCA = right coronary artery

Table S2

Coronary artery changes in 10 untreated and 10 intravenous immunoglobulin-treated Kawasaki disease patients (these samples were used for validation of miRNA microarray analysis results by real-time quantitative PCR)

**Patient**	**Age**	**Sex**	**Treatment**	**Coronary Change**
Patient 0	1Y2M	female	- IVIG	LCA=3.3 mm
Patient 1	2Y10M	male	- IVIG	LCA=2.4 mm RCA=2.3 mm
Patient 25	1Y10M	male	- IVIG	LCA=3.0 mm RCA=2.1 mm
Patient 50	10M	male	- IVIG	LCA=2.3 mm RCA=2.4 mm
Patient 52	3Y	male	- IVIG	LCA=3.2 mm RCA=2.6 mm
Patient 23	2Y4M	male	- IVIG	LCA=3.0 mm
Patient 73	1Y	female	- IVIG	LCA=3.0 mm RCA=2.5 mm
Patient 88	1Y11M	male	- IVIG	LCA=3.2 mm RCA=3.0 mm
Patient 111(0)	1Y3M	male	- IVIG	Coronary artery intimal hyperplasia
Patient 111(2)	1Y3M	male	- IVIG	Coronary artery intimal hyperplasia
Patient 17	1Y8M	male	+ IVIG	LCA=2.7 mm RCA=2.4 mm
Patient 21	1Y4M	male	+ IVIG	LCA=2.5 mm RCA=2.1 mm
Patient 29	10M	female	+ IVIG	LCA=3.3 mm RCA=2.4 mm
Patient 64	3Y	male	+ IVIG	LCA=2.6mm RCA=2.2mm
Patient 65	11M	male	+ IVIG	LCA=2.3 mm RCA=2.2 mm
Patient 66	8M	female	+ IVIG	LCA=2.5 mm RCA=2.9 mm
Patient 74	11M	male	+ IVIG	LCA=2.3 mm RCA=2.2 mm
Patient 79	1Y	male	+ IVIG	LCA=2.5 mm RCA=2.0 mm
Patient 89	1Y	female	+ IVIG	LCA=2.8 mm RCA=2.9 mm
Patient 111(2)	1Y3M	male	+ IVIG	Coronary artery intimal hyperplasia

Y = year; M = month; KD = Kawasaki disease; IVIG = intravenous immunoglobulin; -IVIG = KD patients before IVIG therapy; +IVIG = KD patients after IVIG therapy; LCA = left coronary artery; RCA = right coronary artery

Table S3

Coronary artery changes in 10 Kawasaki disease patients before and after intravenous immunoglobulin therapy (these samples were used for validation of miRNA microarray analysis results by real-time quantitative PCR)

**Patient**	**Age**	**Sex**	**Treatment**	**Coronary Change**
Patient 3	11M	male	- IVIG	LCA=3.1 mm RCA=3.0 mm
			+ IVIG	LCA=2.7 mm RCA=2.6 mm
Patient 36	2Y11M	female	- IVIG	LCA=4.2 mm
			+ IVIG	LCA=3.3 mm RCA=2.4 mm
Patient 63	10M	male	- IVIG	LCA=5.2 mm RCA=7.8 mm CAA
			+ IVIG	LCA=5.0 mm RCA=7.6 mm CAA
Patient 71	2Y	male	- IVIG	LCA=2.5 mm RCA=2.4 mm
			+ IVIG	LCA=2.5 mm RCA=2.3 mm
Patient 80	1Y	male	- IVIG	LCA=2.6 mm RCA=2.0 mm
			+ IVIG	LCA=2.4 mm RCA=1.9 mm
Patient 102	5Y	male	- IVIG	LCA=3.3 mm RCA=3.7 mm
			+ IVIG	LCA=3.1 mm RCA=2.9 mm
Patient 104	2M	male	- IVIG	LCA=3.1 mm RCA=2.5 mm
			+ IVIG	LCA=3.0 mm RCA=2.2 mm
Patient 125	1Y10M	female	- IVIG	LCA=3.3 mm RCA=2.3 mm
			+ IVIG	LCA=3.0 mm RCA=2.2 mm
Patient 129	1Y7M	male	- IVIG	LCA=3.2 mm RCA=3.1 mm
			+ IVIG	LCA=2.9 mm RCA=2.8 mm
Patient133	1Y4M	female	- IVIG	LCA=3.2 mm RCA=2.0 mm
			+ IVIG	LCA=2.8 mm RCA=2.0 mm

Y = year; M = month; KD = Kawasaki disease; IVIG = intravenous immunoglobulin; -IVIG = KD patients before IVIG therapy; +IVIG = KD patients after IVIG therapy; LCA = left coronary artery; RCA = right coronary artery; CAA = coronary artery aneurysm

Table S4

Coronary artery changes in 54 Kawasaki disease patients before IVIG therapy (these samples were used for validation of the biomarker candidates for Kawasaki disease diagnosis by real-time quantitative PCR)

Patient	Age	Sex	Diagnosis	Coronary Change
Patient 32	3Y	male	KD	LCA=2.7 mm RCA=2.3 mm
Patient 59	8M	female	KD	LCA=2.0 mm RCA=2.6 mm
Patient 101	2Y6M	male	KD	LCA=2.4mm RCA=2.4mm
Patient 105	1Y3M	male	KD	Coronary artery intimal hyperplasia
Patient 106	2Y7M	female	KD	Coronary artery intimal hyperplasia
Patient 114	2Y4M	male	KD	Coronary artery intimal hyperplasia
Patient 116	3M	male	KD	Coronary artery intimal hyperplasia
Patient 119	2Y2M	female	KD	Coronary artery intimal hyperplasia
Patient 123	5M	male	KD	LCA=3.5 mm RCA=2.2 mm
Patient 129	1Y7M	male	KD	LCA=3.2 mm RCA=3.1 mm
Patient 132	2Y2M	male	KD	LCA=2.6mm RCA=2.2
Patient 135	1Y1M	female	KD	Coronary artery intimal hyperplasia
Patient 136	8M	male	KD	Coronary artery intimal hyperplasia
Patient 140	2Y1M	female	KD	LCA=3.0 mm RCA=3.1 mm
Patient 143	3M	female	KD	Coronary artery intimal hyperplasia
Patient 145	7Y	male	KD	Coronary artery intimal hyperplasia
Patient 147	2Y4M	male	KD	Coronary artery intimal hyperplasia
Patient 149	2Y2M	male	KD	Coronary artery intimal hyperplasia
Patient 150	6M	male	KD	Coronary artery intimal hyperplasia
Patient 151	1Y1M	male	KD	LCA=2.9 mm RCA=2.1 mm
Patient 171	4M	male	KD	LCA=3.3 mm RCA=2.8 mm
Patient 172	2Y6M	male	KD	LCA=3.7 mm RCA=3.9 mm
Patient 173	2M	male	KD	LCA=2.6 mm RCA=2.1 mm
Patient 174	1Y1M	male	KD	LCA=3.2 mm RCA=2.5 mm
Patient 176	3M	female	KD	LCA=2.3 mm RCA=1.8 mm
Patient 177	1Y1M	male	KD	LCA=2.8 mm RCA=2.4 mm

Patient 178	4Y	male	KD	LCA=3.8 mm RCA=2.7 mm
Patient 179	4M	male	KD	LCA=3.6 mm RCA=2.9 mm
Patient 181	2Y7M	male	KD	LCA=2.8 mm RCA=3.7 mm
Patient 182	3M	female	KD	LCA=3.5 mm RCA=3.9 mm
Patient 183	5Y	female	KD	LCA=3.1 mm
Patient 186	10Y	male	KD	LCA=4.4 mm RCA=4.2 mm
Patient 187	3Y	male	KD	LCA=3.5 mm RCA=3.6 mm
Patient 188	7M	male	KD	LCA=2.5 mm RCA=2.0 mm
Patient 189	4Y	female	KD	LCA=3.1 mm RCA=2.2 mm
Patient 190	7M	male	KD	LCA=3.0 mm RCA=2.4 mm
Patient 191	2Y8M	male	KD	LCA=3.9 mm
Patient 212	1Y3M	male	KD	LCA=3.2mm RCA=2.1mm
Patient 213	3Y	male	KD	LCA=3.8mm RCA=2.3mm
Patient 215	1Y1M	male	KD	LCA=3.2mm RCA=2.0mm
Patient 218	1Y11M	male	KD	LCA=6.8mm RCA=4.7mm
Patient 229	11M	male	KD	LCA=3.7mm RCA=2.0mm
Patient 232	1Y10M	male	KD	LCA=3.3mm RCA=2.0mm
Patient 233	1Y7M	male	KD	LCA=2.8mm RCA=2.5mm
Patient 234	4Y	male	KD	LCA=4.0mm RCA=3.6mm
Patient 236	1Y1M	male	KD	LCA=3.5mm RCA=2.2mm
Patient 237	7M	male	KD	LCA=2.7mm
Patient 238	1Y2M	male	KD	LCA=3.0mm RCA=2.2mm
Patient 239	1Y8M	male	KD	LCA=2.7mm RCA=3.0mm
Patient 2-10	1Y6M	male	KD	Coronary artery intimal hyperplasia
Patient 2-12	1Y9M	female	KD	Coronary artery intimal hyperplasia
Patient 2-14	1Y8M	male	KD	Coronary artery intimal hyperplasia
Patient 2-15	1Y	female	KD	Coronary artery intimal hyperplasia
Patient 2-16	2Y8M	male	KD	Coronary artery intimal hyperplasia

Y = year; M = month; KD = Kawasaki disease; IVIG = intravenous immunoglobulin; LCA = left coronary artery; RCA = right coronary artery

Table S5

The list of All RT-PCR primer sequences (5'-3')

miRNA	*Reverse transcription primers*
hsa-miR-4436b-5p	GTCGTATCCAGTGCTGGGTCCGAGTGATTCGCACTGGATACGACGGCAGG
hsa-miR-671-5p	GTCGTATCCAGTGCTGGGTCCGAGTGATTCGCACTGGATACGACCTCCAG
hsa-miR-1246	CTCAACTGGTGTCGTGGAGTCGGCAATTCAGTTGAGCCTGCTCC
hsa-miR-197-3p	CTCAACTGGTGTCGTGGAGTCGGCAATTCAGTTGAGGCTGGGTG

miRNA	*qPCR forward primer*	*qPCR reverse primer*
hsa-miR-4436b-5p	AGCCCGTCCACTTCTGCC	CAGTGCTGGGTCCGAGTGA
hsa-miR-671-5p	GCCGAGAGGAAGCCCTGG	CAGTGCTGGGTCCGAGTGA
hsa-miR-1246	ACACTCCAGCTGGGAATGGATTTTTGGAGC	CTCAACTGGTGTCGTGGA
hsa-miR-197-3p	ACACTCCAGCTGGGTTCACCACCTTCTCCACC	CTCAACTGGTGTCGTGGA
